# Theoretical and Experimental Spectroscopic Analysis of Cyano-Substituted Styrylpyridine Compounds

**DOI:** 10.3390/ijms14024005

**Published:** 2013-02-18

**Authors:** Maria Eugenia Castro, Maria Judith Percino, Victor M. Chapela, Margarita Ceron, Guillermo Soriano-Moro, Jorge Lopez-Cruz, Francisco J. Melendez

**Affiliations:** 1 Chemistry Center, Institute of Sciences, Autonomous University of Puebla, Complex of Sciences, ICUAP, 103H, 22 Sur y San Claudio, University City, Puebla City, Pue.-72570, Mexico; E-Mails: mareug.castro@correo.buap.mx (M.E.C.); judith.percino@correo.buap.mx (M.J.P.); victor.chapela@correo.buap.mx (V.M.C.); margarita.ceron@correo.buap.mx (M.C.); jesus.soriano@correo.buap.mx (G.S.-M.); 2 Theoretical Chemistry Laboratory, Investigation Center, Department of Physical Chemistry, Faculty of Chemical Sciences, Autonomous University of Puebla, 105I, San Claudio y 22 Sur, University City, Puebla City, Pue.-72570, Mexico; E-Mail: jorge.lc@gmail.com

**Keywords:** styrylpyridine compounds, DFT calculations, IR spectroscopy, UV-Vis spectroscopy, NMR spectroscopy

## Abstract

A combined theoretical and experimental study on the structure, infrared, UV-Vis and ^1^H NMR data of *trans*-2-(*m*-cyanostyryl)pyridine, *trans*-2-[3-methyl-(*m-*cyanostyryl)] pyridine and *trans*-4-(*m*-cyanostyryl)pyridine is presented. The synthesis was carried out with an efficient Knoevenagel condensation using green chemistry conditions. Theoretical geometry optimizations and their IR spectra were carried out using the Density Functional Theory (DFT) in both gas and solution phases. For theoretical UV-Vis and ^1^H NMR spectra, the Time-Dependent DFT (TD-DFT) and the Gauge-Including Atomic Orbital (GIAO) methods were used, respectively. The theoretical characterization matched the experimental measurements, showing a good correlation. The effect of cyano- and methyl-substituents, as well as of the *N*-atom position in the pyridine ring on the UV-Vis, IR and NMR spectra, was evaluated. The UV-Vis results showed no significant effect due to electron-withdrawing cyano- and electron-donating methyl-substituents. The *N*-atom position, however, caused a slight change in the maximum absorption wavelengths. The IR normal modes were assigned for the cyano- and methyl-groups. ^1^H NMR spectra showed the typical doublet signals due to protons in the *trans* position of a double bond. The theoretical characterization was visibly useful to assign accurately the signals in IR and ^1^H NMR spectra, as well as to identify the most probable conformation that could be present in the formation of the styrylpyridine-like compounds.

## 1. Introduction

Low molecular weight and oligomeric organic compounds with optical or electrical properties have been widely used as dyes in organic electronic devices, including organic light emitting diodes (OLEDs), solar cell organic semiconductor lasers, *etc.* [1–6]. Typically, these dyes have a donor (D) −π–acceptor (A) structure that controls their photophysical properties [2,7,8]. On the other hand, it is well know that cyano-substituted compounds show good optical and electrical properties due to their high electron affinities [9]. In particular, some cyano-substituted compounds have been reported, which show unique enhanced emission rather than fluorescence quenching in the solid state [10]. However, pyridine is an important electron-acceptor group, due to its high electron affinity. Dailey S. *et al.* described poly(2,5-pyridinediyl) (PPY) as an efficient electron transport layer in bilayer polymeric LEDs. The OLEDs with a PPY layer exhibited external quantum efficiency 60-times greater than those of similar devices without a PPY layer [11]. Epstein *et al.* described poly(*p*-pyridine)- and poly(*p*-pyridyl vinylene)-based polymers as emissive layers in light-emitting devices [12]. Bartholomew *et al.* [13] studied model compounds, such as phenylated PPV, also with the electron withdrawing cyano group attached to the olefin, and they can also be used as a model cyano substitute (CN-PPV).

On the other hand, the effect of introducing nitrogen atoms in the aromatic rings of *trans*-1,2-diarylethylenes has been widely investigated in order to study the effect of heteroatoms on the excited state properties of stilbene, related diarylethenes and *n*-styrylpyridines [14–16]. Model compounds in *trans* conformation or with the group CN attached to double bond or in the aromatic ring [17–21] have been studied to comprehend the effect in the optics and electronic properties. One of our studies on styrylpyridines derivatives was on 2,6-distyrylpyridine [22,23]. The characterization results by X-ray showed that although the molecule is plane, it did not exhibit a total delocalization of its electrons through the whole molecule. Also, the results showed that depending on the *trans* spatial arrangement of double bonds, there were three possible conformations. The theoretical calculations reported showed the conformational and structural analyses of 2,6-distyrylpyridine conformations using *ab initio* methods, as well as methods based on density functional theory, DFT [23]. The results determined that the conformation depends on the double bond orientation by the *trans* proton steric effect with protons in the *ortho* position of the phenyl or with pyridine groups. For our study, these molecules are chosen, because they contain a cyano group as a strong acceptor attached to the aromatic ring, a pyridine group as a weak acceptor (in *o*- and *p*-position) through double bonding and a methyl moiety as a donor by the inductive effect, to evaluate which kind of structure, D–π–A or A–π–A, is present.

Several styrylpyridine-like compounds—2-styrylpyridine, its derivatives and intermediates [24–27], 4-styrylpyridine and its intermediates [27–29], 2,6-distyrylpyridine [23,27], pyridinevinylenes [30] and phenylpyridylacrylonitriles [19]—have been synthesized under green chemistry conditions. Some have been characterized by X-ray diffraction [22,24–29], IR [19,22,25–30], ^1^H-NMR [19,24–30], UV-Vis [19,28], mass spectroscopy [19,27] and complementary theoretical studies [23,31]. Theoretical calculations on 2,6-distyrylpyridine [23,31], 4-styrylpyridine [32,33] and 2-styrylpyridine and derivatives [34] have been carried out using *ab initio* and Density Functional Theory (DFT) approximations.

Atalay *et al.* [31] obtained geometric parameters, IR and ^1^H and ^13^C NMR spectra for 2,6-distyrylpyridine using Hartree-Fock (HF) and B3LYP methods with the 6-31G(d) basis set. In this work, the B3LYP calculations fit the experimental data better than HF-derived values in evaluating vibrational frequencies and geometrical parameters [31]. Melendez *et al.* [23], carried out a study of three conformational structures of 2,6-distyrylpyridine using HF, second-order Moller-Plesset (MP2) and B3LYP methods with 6-31G(d,p) and cc-pVDZ basis sets. The authors indicated that B3LYP and MP2 methods with the cc-pVDZ basis set would be good choices for structural, stability and reactivity analyses for these kinds of compounds [23]. Daku *et al.* [32,33] analyzed the 4-styrylpyridine *cis* and *trans* isomers using HF, MP2 and coupled cluster (CC) *ab initio* methods, DFT and Molecular Dynamics (MD) methods. Similar results were found for the geometry and energy determination using these different methods, allowing the authors to predict the molecular structure as planar at the energy minimum corresponding to the *trans* conformation [32]. TD-DFT was used for calculating the absorption spectra of both isomers [33], where the main absorption band was predicted at 314.5 nm, due to the double bond in the *trans* isomer, which originated from the S_0_ to S_1_ transition. On the other hand, *trans*-4-(*m*-cyanostyryl)pyridine was recently studied using DFT methods [34], in order to predict the maximum absorption using the TD-DFT approach. The absorption maximum was calculated to be 307.2 nm, similar to the values obtained by Daku *et al*. [33] and the experimental value of 304 nm [30] for 4-styrylpyridine. Castro *et al.* [34] also reported that the calculated values for the maximum absorption for the 2-styrylpyridine and cyano- and methyl-substituted derivatives by DFT methods were close to experimentally determined values.

DFT calculations, specifically hybrid methods, namely B3LYP, PBE and PBE0, have been shown to be a good approximation method for providing accurate molecular parameters, infrared spectrum assignment, ^1^H chemical shifts and UV-Vis spectrum, and are in good agreement with experimental data for styrylpyridine-like compounds [23,31–34] and other organic conjugated compounds [35–39].

In this work, we present the synthesis and spectroscopic characterization of three cyano-substituted styrylpyridine compounds, which were obtained using green chemistry conditions. These structures are derivatives of 2- and 4-styrylpyridine with the –C≡N group attached at the *meta* position of the phenyl ring. These compounds were characterized theoretical and experimentally by IR, ^1^H NMR and UV-Vis spectroscopy. Optimized structural parameters were obtained using the Density Functional Theory (DFT), performing B3LYP/6-311 + G(d,p) theoretical calculations in both gas and solution phases [34]. Infrared frequencies (IR) spectra, UV-Vis transitions and ^1^H NMR shifts were obtained. Our analyses of these soluble styrylpyridine model compounds allowed us to gain insights into the role of the substituents and position of the *N*-atom in determining the spectroscopic characteristics of this class of compounds.

## 2. Computational Methodology

**Figure 1:**
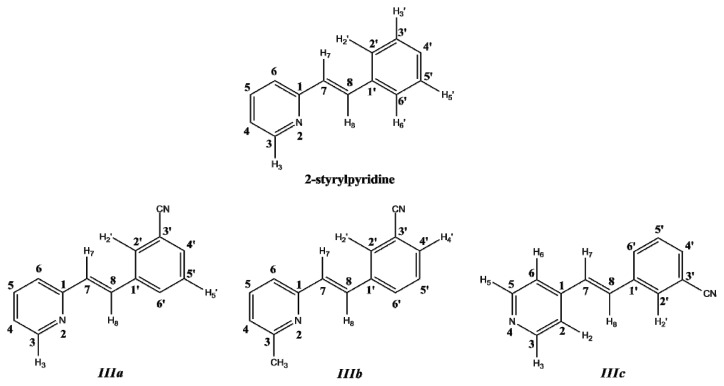
Molecular structure and numerical conventions of *trans*-2-(*m*-cyanostyryl)pyridine (***IIIa***), *trans*-2-[3-methyl-(*m*-cyanostyryl)]pyridine (***IIIb***) and *trans*-4-(*m*-cyanostyryl) pyridine (***IIIc***).

The three styrylpyridine-like model compounds were investigated by DFT theoretical calculations. The molecular structures and the numerical conventions used for carrying out the theoretical calculations are shown in Figure 1.

The optimization calculations in gas and solution phases were performed by the DFT method taking into account the electron correlation contribution, which is especially important in conjugated systems. B3LYP functional [40,41] with the 6-311 + G(d,p) basis set [42] was used. The solvent effect of CHCl_3_ (with the dielectric constant ɛ = 4.71) was included by using the Polarizable Continuum Model (PCM) [43].

Vibrational frequencies were calculated at the same theory level as the optimization calculations to ensure the minima structures of the compounds and to obtain the vibrational analysis using the scale factor of 0.9648 [44]. Electronic transition energies were calculated by the TD-B3LYP/6-31G(d) method [45–48] from the B3LYP/6-311 + G(d,p) optimized geometries to reproduce UV-Vis spectroscopic transitions. Finally, ^1^H NMR chemical shifts were obtained by the Gauge-Independent Atomic Orbital (GIAO) approach [49,50] in the B3LYP/6-31 + G(d,p)//B3LYP/6-311 + G(d,p) level. All the calculations were carried out in the Gaussian 09 program [51].

## 3. Results and Discussion

### 3.1. Synthesis

**Figure 2:**
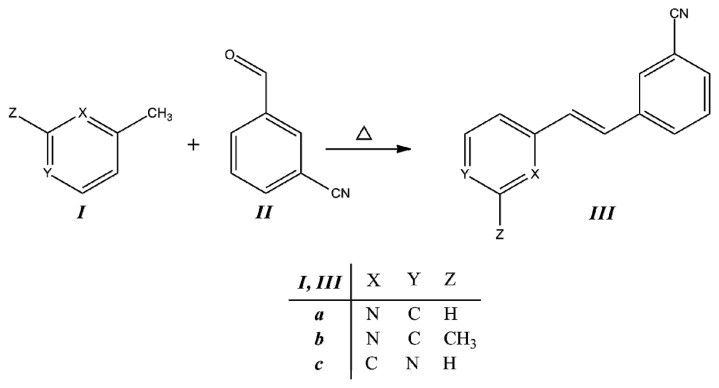
Synthesis of *trans*-2-(*m*-cyanostyryl)pyridine (***IIIa***), *trans*-2-[3-methyl-(*m-*cyanostyryl)] pyridine (***IIIb***) and *trans*-4-(*m*-cyanostyryl)pyridine (***IIIc***).

Compounds ***IIIa***–***IIIc*** were obtained from the corresponding methylpyridine ***I*** with the corresponding aldehyde ***II***, as is described in the Experimental section. The condensation reaction scheme is presented in Figure 2.

2-Styrylpyridine was synthesized according to the previously reported method [24,27]. The temperature, reaction time and the presence of the –C≡N and –CH_3_ substituents play important roles in the product formation, as well as the presence of either the *ortho*- or *para*-position pyridine. Table 1 shows the yields and measured properties of each compound.

Under these reaction conditions, we obtained ***IIIa***–***IIIc*** in almost the same yield as reported for 2-styrylpyridine [24,27] under the same conditions. Recent studies have described the use of Knoevenagel condensation in the synthesis of several substituted stilbenes in the absence of catalyst and solvent [19,22,24–30]. However, compared with the conditions used for reactions of pyridylacetonitrile with benzaldehyde, we observed that the effect of the –C≡N group on the reactivity was dependent on its site of attachment in the structure. The reactivity was more pronounced for pyridylacetonitrile than for picoline in obtaining the ***IIIa***–***IIIc*** compounds. The reactions conduced without catalyst and solvent gave better yields, even at room temperature [19,30], when the –C≡N group was located on the –CH_2_CN than when located in the aromatic aldehyde. According to the results from the three reactions of Table 1, the reactivity behavior is –CH_3_ moiety < *p*-position < *o*-position on pyridine ring due to the inductive effect. Systems with π–conjugated units provide an effective pathway for the efficient push-pull charge transfer between donor and acceptor groups. Styrylpyridine-like materials are expected to have different applications in photochemistry and fluorescence processes when the donor-acceptor or acceptor-acceptor is introduced in the phenyl rings in order to extend the conjugation over the whole molecule.

### 3.2. Molecular Structures

Optimized parameters were obtained by the B3LYP/6-311 + G(d,p) level for the three model compounds. Previous tests were carried out for the isomers of these molecules with the –C≡N group attached to the equivalent phenyl ring in the *meta*-position [34]. The rotamers showed energy values similar to each other (~1 kJ mol^−1^); however, the energy barriers were ~20 to 25 kJ mol^−1^ [34] for the torsion motion of the cyano-substituted phenyl ring. The energetically most stable isomers of molecules ***IIIa***–***IIIc*** were those with the –C≡N group attached in position C(3′). Molecules ***IIIa*** and ***IIIb*** displayed an orientation *anti* to the –C≡N group with respect to the *N*-atom of the pyridine ring, but for ***IIIc***, the orientation was different (Figure 1).

**Figure 3:**
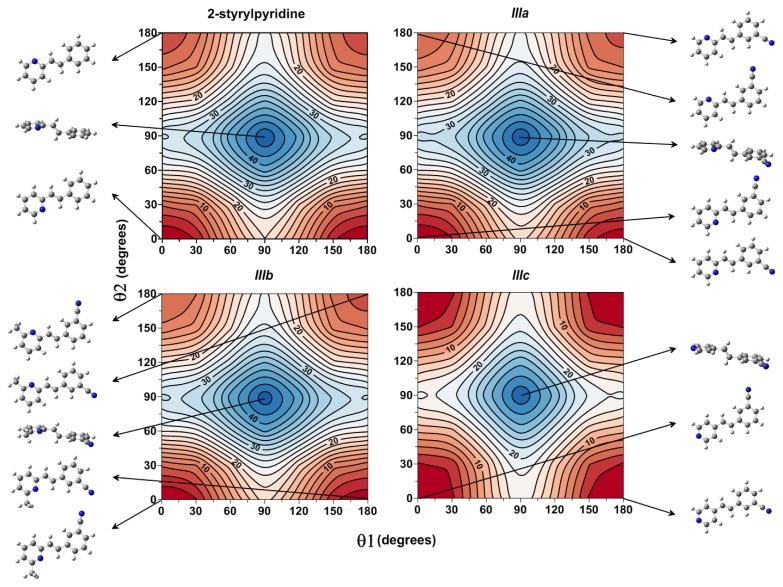
Isocontour potential energy maps as a function of θ1 and θ2 torsional angles of 2-styrylpyridine, *trans*-2-(*m*-cyanostyryl)pyridine (***IIIa***), *trans*-2-[3-methyl-(*m-*cyanostyryl)] pyridine (***IIIb***) and *trans*-4-(*m*-cyanostyryl)pyridine (***IIIc***) obtained at the B3LYP/6-311 + G(d,p) theory level. The structures at the minima and maximum are included.

In this work, a two-dimensional conformational analysis was carried out for evaluating simultaneously the effect of the torsion of phenyl group, θ1, and the pyridine group, θ2. The two conformational coordinates were defined as dihedrals θ1, between atoms C(7)–C(8)–C(1′)–C(2′) for 2-styrylpyridine, ***IIIa*** and ***IIIb*** and C(7)–C(8)–C(1′)–C(6′) for ***IIIc*** and θ2, between atoms N(2)–C(1)–C(7)–C(8) for 2-styrylpyridine and ***IIIa*** and ***IIIb*** and C(2)–C(1)–C(7)–C(8) for ***IIIc*** (Figure 1). For the four molecules, a grid of points was generated on each conformational coordinate using increments of 30° in a range of 0°–180°. At each grid point, the conformational coordinates were kept frozen, whereas the rest of the structure was fully relaxed. Figure 3 shows the isocontour potential energy maps as the function θ1 and θ2 torsional angles described. The interval between isocontour lines is 10 kJ mol^−1^, and the data refer to the minimum value. Red zones correspond to lower energy zones, whereas blue zones correspond to higher energy zones. The structures of the minima and maximum are included in the figure.

Figure 3, for the case of 2-styrylpyridine, shows double minima arising from the symmetric torsion of the phenyl ring; therefore, equivalent minima for (θ1, θ2) (0°, 0° = 180°, 0° and 0°, 180° = 180°, 180°) are found. The global minimum (0°, 0°) and the local minimum (0°, 180°) were located on the potential energy map for the torsion of the pyridine group with a relative energy between rotamers of 4.65 kJ mol^−1^; see Table 2. For ***IIIa*** and ***IIIb***, in addition to the minima due to the torsion of the pyridine ring, another two minima were located due to the torsion of the cyano-substituted phenyl. So, the rotamer (180°, 0°) is almost energetically equivalent to global minimum (0°, 0°) in less than 1 kJ mol^−1^, while two local minima in (0°, 180°) and (180°, 180°) were found with energy differences of about 1 kJ mol^−1^ between them (Table 2). For ***IIIc***, the position of the *N*-atom in the pyridine ring provided two symmetric minima with respect to the pyridine torsion, while the global minimum was found for the (0°, 180°) and the local minimum for (0°, 0°) rotamers. For the four molecules, the activation barriers were found at ~34 to 46 kJ mol^−1^, corresponding to structures with (θ1, θ2) = (90°, 90°).

In order to complement the conformational study, which provides results based essentially on enthalpy values, we achieved a thermostatistical analysis to account for entropic effects. The population of the different rotamers was calculated at room temperature by using the technique developed by Niño *et al.* [52]. The populations (in percent) were calculated from the relative energies obtained at the B3LYP/6-311 + G(d,p) theory level in Figure 3. The results collected in Table 2 show that for 2-styrylpyridine, the population for the global minimum is considerably larger (11.85%) than that for the local minimum (1.80%), confirming that the rotamer that is energetically more stable (0°, 0°) is the most populated. For ***IIIa*** and ***IIIb***, it was found that the minima (0°, 0°) and (180°, 0°) energetic equivalents had populations with values of less than 1.5% of the difference between them, while the minima corresponding to the rotamers (0°, 180°) and (180°, 180°) gave small population values (Table 2). Finally, for ***IIIc***, the difference in population between the global and local minima is less than 1%. In all cases, the rotamers with maximum energies gave a population of 0%, as was expected.

The results mentioned showed the minima (0°, 0°) and (180°, 0°) of ***IIIa*** and ***IIIb*** can exist in equivalent proportions, demonstrating that different isomer states can have similar populations.

On the other hand, the compounds involving symmetric groups in their structures, 2-styrylpyridine and ***IIIc***, showed opposing behaviors. For example, in 2-styrylpyridine, the global and local minima, arising from the torsion of pyridine, had conformational energies and populations larger than in ***IIIc***, in which both minima arise from the torsion of the cyano-substituted phenyl with similar values between them.

Selected optimized parameters in the gas phase and those, including the solvent CHCl_3_ effect, for model styrylpyridine-like compounds ***IIIa***–***IIIc*** are summarized in Table 3.

B3LYP/6-311 + G(d,p) has been shown to be an adequate level of theory for the geometry calculation for molecules of this kind [34]. The internuclear distances, valence angles and dihedral angles did not show significant changes among these three molecules. No important differences were found between the gas phase values and those with the solvent effect for the three molecules (Table 3). Three compounds showed planar structures according to the X-ray data of the title compounds 2-styrylpyridine [24] and 4-styrylpyridine [53].

### 3.3. IR Spectroscopy

Theoretical IR spectra of 2-styrylpyridine and ***IIIa***–***IIIc*** compounds showed similar characteristic infrared band frequencies in the gas phase and when including the solvent CDCl_3_ effect. Theoretical scaled frequencies were obtained by using the scale factor of 0.9648 [44]. The results of the calculated and experimental harmonic frequencies are collected in Table 4.

Of the ring vibrations, the CH bond stretching of the aromatic ring appeared at the 3100.0–3000.0 cm^−1^ region. The ν(C–H) mode for pyridine was found in the range of 3079.4–3034.2 cm^−1^ in the gas phase and at the 3081.3–3036.2 cm^−1^ range in the solution phase. The ν(C–H) mode for the phenyl ring was calculated at 3090.5–3078.8cm^−1^ and 3093.3–3080.1 cm^−1^ ranges in gas and solution phases, respectively, (Table 4). The experimental values at 3074–3032 cm^−1^ for ν(C–H)Py and at 3154–3079 cm^−1^ for ν(C–H)Ph were in accord with an earlier report for aromatic compounds [54].

Spectral IR characterization of halogen-substituted compounds showed bands between 3004 and 3076 cm^−1^ for ν(C–H) ring vibrations [30]. These ranges were in good agreement with the values obtained for our cyano-substituted 2-styrylpyridine (***IIIa*** and ***IIIb***) and 4-styrylpyridine (***IIIc***).

Stretching modes ν(C=C) and ν(C=N) of the pyridine ring were calculated at 1550.6–1390.2 cm^−1^ and 1576.6–1446.8 cm^−1^, respectively, in the gas phase, whilst values of 1546.7–1389.7 cm^−1^ and 1574.2–1446.5 cm^−1^ were calculated in the solution phase (Table 4). In the experimental IR spectrum, the bands at the 1580–1418 cm^−1^ range were also assigned to these stretching modes due to ν(C=C)Py and ν(C=N)Py of the pyridine ring (1600–1430 cm^−1^ range) [55].

For 2-styrylpyridine and ***IIIa***–***IIIc*** compounds, a combination of bands resulting from the interaction between two vibrations were found coupled in the 1544.0–1471.9 cm^−1^ and 1541.6–1470.6 cm^−1^ regions corresponding to the stretching modes [ν(C=N) + ν(C=C)] for the pyridine, in the gas, as well as the solution phase. On the other hand, coupled stretching modes ν(C=N) of pyridine + ν(C=C) of phenyl were found in the 1578.0–1556.1 cm^−1^ and 1575.9–1553.7 cm^−1^ regions in both phases (Table 4). In the 1600–1500 cm^−1^ region [54] the most of the six-membered aromatic ring systems is reported.

In contrast, the characteristic ν(C=C) mode of the alkene double bond when it is conjugated with aromatic rings was obtained at 1628.9–1625.9 cm^−1^ and 1626.8–1624.1 cm^−1^, in gas and solution phases, respectively, and the experimental measurement appeared at 1641–1635 cm^−1^. These values were in accord with 1625 cm^−1^ reported [55] for this mode.

The band in the regions of 981.5–968.3 cm^−1^ and 982.5–972.3 cm^−1^ was assigned to the vibration associated with the out of plane deformation δ(C–H) due to the protons of a double bond –CH=CH– in the *trans* configuration, as calculated in both gas and solution conditions. The experimental values appeared at the 985–973 cm^−1^ region, in good agreement with the calculated values and with the values of 980–960 cm^−1^ reported [55], as well as with the values reported previously for 2- and 4-styrylpyridines with halogen substituents [30].

The mode ν(C≡N) for the cyano-substituted styrylpyridine compounds ***IIIa***–***IIIc*** was found at 2252.3–2250.3 cm^−1^ and 2242.5 cm^−1^ in the gas and solution phases, respectively. The experimental spectra for ***IIIa***–***IIIc*** compounds showed the characteristic band shape in the 2232–2230 cm^−1^ range, which is the characteristic vibration frequency value for this mode assigned to compounds containing a cyano moiety [54].

For compound ***IIIb***, the band corresponding to the CH stretching modes of the –CH_3_ appeared at 2982.6–2928.2 cm^−1^ region in gas phase and the 2980.0–2926.9 cm^−1^ region in solution phase, and the experimental values were two bands at 2959 and 2925 cm^−1^. These values agreed with the experimental value reported of 2960 cm^−1^ for the asymmetric stretching and 2870 cm^−1^ for the symmetric one [56].

Alternatively, out-of-plane CH deformations for the methyl group for the compound ***IIIb*** were found in the ranges of 1020.9–1019.8 cm^−1^ and 1446.0–1439.6 cm^−1^ for the asymmetric flexions δ^−^_as_(CH_3_) and δ^+^_as_(CH_3_), respectively, whilst the symmetric bending δ_s_(CH_3_) was found at 1360.8–1356.7 cm^−1^. These values matched with the values of 1375 and 1450 cm^−1^, respectively, for symmetric and asymmetric flexions reported in the literature [55] and in agreement with the observed values for the ***IIIb*** spectrum of 1374 and 1451 cm^−1^, respectively.

### 3.4. UV Spectroscopy

The electronic transition energies have been calculated within the framework of the Time-Dependent Density Functional Theory method (TD-DFT) [45–48]. These calculations have been performed on the lowest energy structures of each model compound as obtained from B3LYP/6-311 + G(d,p) calculations in gas phase and including the solvent CHCl_3_ effect. The TD-DFT approach accounts for the dynamic electron correlation caused by the coupling with the correlated ground state function. A previous comparative study [34] using ZINDO/S, TD-B3LYP and TD-PBE0 methods with different basis sets showed that the theoretical λ_max_ bands related to the electronic transition between S_0_→S_1_ states were more exact, as compared with the available experimental data, using the TD-B3LYP/6-31G(d,p)//B3LYP/6-311 + G(d,p) theory level in gas phase.

The results for the electronic transitions, their assignments, the maxima absorption and the oscillator strengths of the styrylpyridine-like model compounds in the gas phase and including the solvent CHCl_3_ effect are shown in Table 5. The major MO→MO excitations involved in the three lowest-lying transitions are also indicated.

The experimental maximum absorptions, λ_max_, were observed at 311.0, 309.6, 312.1 and 293.6 nm for 2-styrylpyridine and molecules ***IIIa***–***IIIc***, respectively (Table 5). The TD-B3LYP/6-31G(d,p)//B3LYP/6-311 + G(d,p) calculations carried out correctly predicted the maximum absorption wavelengths, λ_max_ (nm), with errors of 0.19%, 0.10%, 0.22% and 4.63% in the gas phase. While the values for 2-styrylpyridine, ***IIIa*** and ***IIIb*** were reproduced correctly, the λ_max_ for ***IIIc*** was shifted ~14 nm toward the shorter wavelength region of the spectrum in the experimental data. In the solution phase, similar errors of 3.95%, 3.64% and 3.87% were found for 2-styrylpyridine and ***IIIa*** and ***IIIb***, respectively, while a considerable increment of 8.41% was obtained for ***IIIc***.

The results in Table 5 indicated that the maximum absorption wavelength for the 2-styrylpyridine and ***IIIa***–***IIIc*** compounds are mainly associated with the S_0_–S_1_ transition, with the largest oscillator strength values. This transition involves the promotion of one electron from the bonding highest-occupied MO (HOMO) into the antibonding lowest-unoccupied MO (LUMO), *i.e.*, it is due to the π→π* transition in the *trans* configuration. In the case of 2-styrylpyridine, the contribution of the HOMO→LUMO excitation was 98% for the S_0_–S_1_ transition, while for the S_0_–S_2_ transition, the main contribution (97%) was derived from the excitation of HOMO-2→LUMO. The S_0_–S_3_ transition in 2-styrylpyridine was characterized by two excitations contributed by HOMO-1→LUMO (74%) + HOMO→LUMO + 2 (25%). For the cyano-substituted compounds, the excitation of HOMO→LUMO was the main contribution to the first S_0_–S_1_ transition (98%, 97% and 98%, for ***IIIa***, ***IIIb*** and ***IIIc***, respectively). The excitation of HOMO-1→LUMO contributed 96% to the second lowest-lying transition S_0_–S_2_ in the molecules ***IIIa*** and ***IIIb***. The third lowest-lying transition S_0_–S_3_, corresponding to the HOMO→LUMO + 1 + HOMO-2→LUMO excitations, contributed 86% and 12%, respectively, in ***IIIa*** and 82% and 14%, respectively, in ***IIIb***. For compound ***IIIc***, the HOMO→LUMO+1 (71%) + HOMO-3→LUMO (24%) and HOMO-1→LUMO (93%) excitations made the greatest contributions, the second and third lowest-lying transitions, respectively. This behavior explains the transition bands observed in the experimental spectra for all compounds.

On the other hand, Percino *et al.* recently reported absorption spectra of Cl- and F-substituted styrylpyridines that showed one strong absorption signal in the range of 308–318 nm [30], assigned to the π→π* transition for the double bond with substituents in the *trans* position. *ortho*-pyridine vinylene compounds containing –F and –Cl attached in the *meta*-position on the phenyl ring that were analogous to ***IIIa*** showed absorption maxima at 309 and 308 nm, respectively [30]. These authors did not observe a shift of the absorption wavelength owing to the presence of –F or –Cl with respect to the maximum absorption wavelength of 2-styrylpyridine. These results were similar to the value of 309 nm obtained for compound ***IIIa*** containing –C≡N (Table 5). For molecule ***IIIc***, with the *N*-atom position in the *para*-position, we observed hypsochromic shifts of ~16 nm in the experimentally determined spectrum and ~3 nm in the theoretically derived data with respect to ***IIIa***. These results are at odds with the data of Percino *et al.* [30], who reported a red shift in λ_max_ for Cl-substituted 4-styrylpyridine to 318 nm, which indicated a bathochromic effect of the electron-withdrawing halogen substituent.

Daku *et al.* [33] obtained a value of 314.5 nm for *trans* 4-styrylpyridine by using LR-TDDFT calculations for the first excited state S_0_–S_1_ with an oscillator strength value of 0.90418. This value is approximate to that obtained in the present work for the *trans* 4-styrylpyridine cyano-substituted (***IIIc***) compound by ~7 nm. In general, the results are in agreement with previous experimental and theoretical results for compounds with a double bond in the *trans* position.

On the other hand, the analysis of the energies of the frontier molecular orbitals and, therefore, the band gap energies, give useful information about the optical and electronic properties of the conjugated compounds. The energies of the main molecular orbitals (HOMO, HOMO-1, LUMO and LUMO + 1 in au) calculated by using the TD-B3LYP/6-311 + G(d,p) theory level in gas and solution phases are collected in Table 6. We also present the calculated HOMO-LUMO gap energies (Δ*E* = Є_LUMO_*−* ª_HOMO_, in eV) of the compounds.

The results indicated similar values for molecular orbital and gap energies for both phases. The electron-withdrawing effects of the cyano group stabilized to the HOMO and LUMO orbitals of ***IIIa***–***IIIc*** with respect to 2-styrylpyridine. Є_HOMO_ and Є_LUMO_ were slightly lower in the gas than in the solution phase, except for 2-styrylpyridine. The smallest gap values were 4.04 and 4.05 eV for 2-styrylpyridine and molecule ***IIIb***, respectively (Table 6). The observation may be due to the inductive effect caused by the electron-donating properties of the –CH_3_ group that counteract the electron-withdrawing effects of the –C≡N.

**Figure 4:**
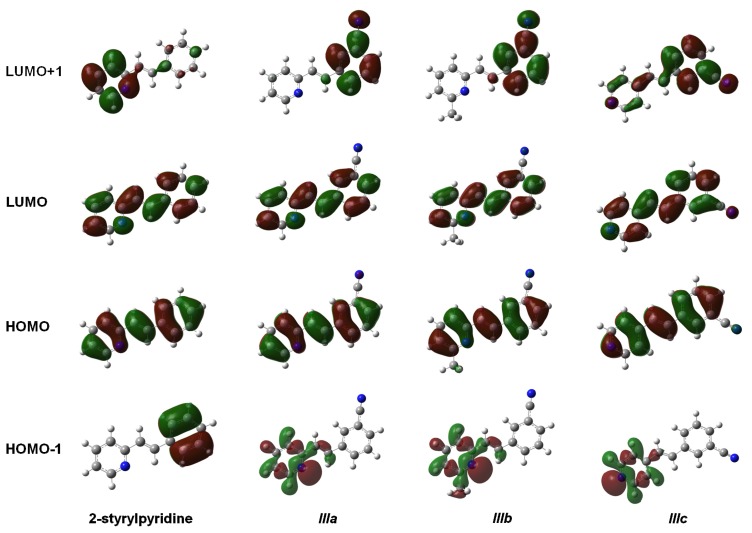
Main molecular orbitals of 2-styrylpyridine, *trans*-2-(*m*-cyanostyryl)pyridine (***IIIa***), *trans*-2-[3-methyl-(*m*-cyanostyryl)]pyridine (***IIIb***) and *trans*-4-(*m*-cyanostyryl)pyridine (***IIIc***) obtained at the TD-B3LYP/6-311 + G(d,p) theory level.

The isosurfaces of the frontier molecular orbitals related to the electronic transition between S_0_→S_1_ states (transition between molecular orbitals HOMO→LUMO) are depicted in Figure 4 for the four molecules at the TD-B3LYP/6-311 + G(d,p) level in the gas phase. Similar orbital plots were obtained when the solvent CHCl_3_ effect was taken into account. In all cases, HOMO and LUMO π MOs were delocalized over the entire molecule. The isosurfaces of the orbital HOMO were localized in four molecules through the central double bond and the double bonds on the rings, whereas the cyano group made a slight contribution to the electronic distribution of the HOMO in molecules ***IIIa***–***IIIc***. In contrast, LUMO distribution was also similar in the four compounds and was localized on the nitrogen and carbon atoms of the rings and on the central carbons in the molecules. This study showed that the methyl group attached to the pyridine ring in molecule ***IIIb*** had no effect on either the HOMO or the LUMO distributions. The electron distributions of the HOMO-1 were not uniform over the whole molecule. In 2-stryrylpyridine, the electron distribution was concentrated in the phenyl ring, but in molecules ***IIIa***–***IIIc***, it was concentrated in the atoms of the pyridine ring, particularly at the lone pair electrons of the *N*-atom. In the case of LUMO + 1, the electron distribution for 2-styrylpyridine was more concentrated on the atoms of the pyridine ring, with a slight concentration on the phenyl ring. The electron distributions for molecules ***IIIa*** and ***IIIb*** were somewhat similar to 2 styrylpyridine, while for ***IIIc***, the density was moved onto the phenyl ring and to a lesser extent onto the double bonds of the pyridine ring (Figure 4).

Thus, through the analysis of UV-Vis spectra, molecular orbital energies and isosurfaces, we are able to make predictions about whether a substituent will cause a blue or a red shift in the maximum absorption peaks, quantitatively and qualitatively, particularly if a compound has the same substituent in the structure.

### 3.5. ^1^H NMR Spectroscopy

The theoretical ^1^H NMR results of the compounds were calculated by using the Gauge-Independent Atomic Orbital (GIAO) method [49,50] at the B3LYP/6-311 + G(2d,p) level from the optimized structures calculated at the B3LYP/6-311 + G(d,p) level in gas phase and the solvent CDCl_3_ effect for the 2-styrylpyridine and the ***IIIa***–***IIIc*** compounds. In order to express the chemical shifts, δ (ppm), the geometry was optimized, and the ^1^H NMR spectrum of the tetramethylsilane (TMS) molecule was calculated using the same method and basis set for use as a reference. The calculated isotropic shielding constants σ_i_ were then transformed to chemical shifts relative to TMS using δ_i_ = σ_TMS_ − σ_I_, where σ_TMS_ = 31.88. The ^1^H NMR data obtained at 400 MHz in CDCl_3_ and theoretical chemical shifts of ^1^H, in ppm, are presented and compared in Table 7.

As it is shown in Table 7, the theoretical δ values were in good agreement with experimental data for the 2-styrylpyridine and the ***IIIa***–***IIIc*** compounds. For 2-styrylpyrydine, ***IIIa*** and ***IIIc***, the δ for H in position 3 (H_3_) was calculated at 8.9 ppm in both gas phase and CDCl_3_ solvent, and the experimental signal was found in 8.6 ppm. Both values agreed very well in the range of chemical shift values for the typical protons for *ortho* position-substituted pyridine compounds at 8.5 ppm [55]. Also, the values were consistent with those reported in the literature for the ring current in Hückel aromatic systems containing heteroatoms, which appeared at 8.59 ppm [57]. On the other hand, the protons of the –CH_3_ at the 3 position for ***IIIb*** (Figure 1) had a calculated δ of 2.7 ppm in both phases and experimentally was a single signal at 2.6 ppm, as typically reported for methyl moieties [57].

The proton attached in position 2′ for 2-styrylpyridine and compounds ***IIIa***–***IIIc*** showed theoretical chemical shifts in agreement with the experimental measurements. Error values calculated with respect to experimental data were 2.9%, 7.8%, 7.6% and 0.1%, respectively, in the gas phase. These error percentages slightly increased by using the PCM model in the calculation. In the case of a proton next to a –C≡N group in mono-substituted benzene, the δ value reported is 7.87 ppm [57]. This value can be roughly compared with δ of H_2′_ proton on the phenyl ring adjoining the –C≡N group in compounds ***IIIa***–***IIIc***; see Figure 1. The results summarized in Table 7, for the theoretical and experimental values obtained in this work, showed that the best approach was for the compound ***IIIc***.

Interesting features were found in the assignment of the protons H_7_ and H_8_. From the ^1^H NMR spectra of all compounds, two doublets corresponding to two protons in the *trans* position with J_H–H_ = 16 Hz were found. It was difficult to determine which signal represented the H_7_ or the H_8_ proton, but the ^1^H NMR calculation results allowed us to assign them adequately. H_7_ and H_8_ protons were experimentally assigned in the 7.2–7.0 ppm and 7.7–7.2 ppm ranges, respectively. The theoretical values were larger than those obtained in the experimental measurements. H_7_ was assigned in a range of approximately 7.6–7.4 ppm, while H_8_ appeared in a range of 8.5–7.4 ppm for the four molecules (Table 7). Using the PCM model, similar ranges were calculated. For H_7_ in gas phase, the theoretical errors with respect to the experimental values were 2.9%, 3.9%, 2.9% and 7.5%, for 2-styrylpyridine and the molecules ***IIIa***–***IIIc***, respectively. Error percentages increased by ~2% for all four molecules by using the PCM model. Errors of 10.1%, 10.4%, 10.5% and 1.8% were obtained for H_8_ in 2-styrylpyridine and ***IIIa***–***IIIc***, respectively, in gas phase, while by using the PCM model, the errors decreased in ~1% for 2-styrylpyridine, ***IIIa*** and ***IIIb***; however, for ***IIIc***, it increased by ~2% with respect to that obtained in the gas phase.

The characteristic chemical shift values for the H_7_ and H_8_ protons attached to a double bond in the *trans* position were influenced toward the lower field due to the extended conjugation caused by the presence of substituents on the aromatic rings [57].

**Figure 5:**
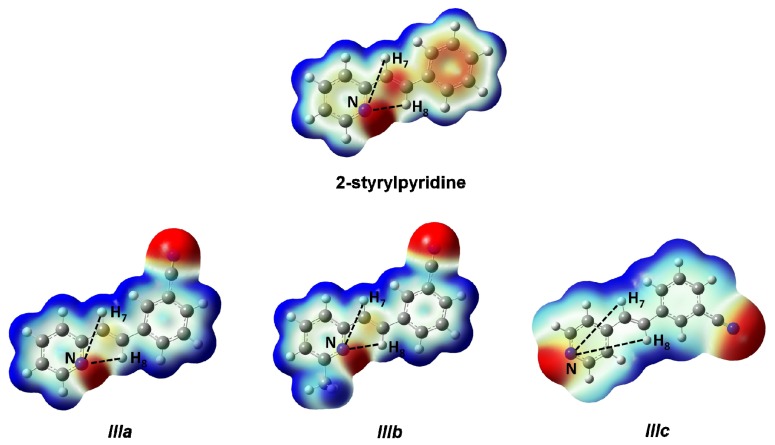
Total electron density mapped with the electrostatic potential of 2-styrylpyridine, *trans*-2-(*m*-cyanostyryl)pyridine (***IIIa***), *trans*-2-[3-methyl-(*m*-cyanostyryl)]pyridine (***IIIb***) and *trans*-4-(*m*-cyanostyryl)pyridine (***IIIc***) obtained at the TD-B3LYP/6-311 + G(d,p) theory level.

Theoretically, the ppm found for the H_7_ and H_8_ protons showed that H_8_ appeared at chemical shifts larger than H_7_ in 2-styrylpyridine and molecules ***IIIa*** and ***IIIb***, which could be explained by the different steric effects due to the presence of the lone pair of electrons at the *N*-atom of the pyridine ring on the H_7_ and H_8_ protons. On the other hand, the electronic density distribution throughout the whole molecule was useful for the correct assignment of δ of these protons. The total electron density mapped with the electrostatic potential surface is presented (isoval = 0.003) in Figure 5. Red regions indicate negative charge, and blue regions indicate positive charge. Yellow regions correspond to an intermediate value between the extremes red and blue.

The distances H_7_–N and H_8_–N were calculated for all four molecules. For 2-styrylpyridine, ***IIIa*** and ***IIIb***, the values were 3.36 and 2.50 Å, respectively. The smaller distance indicates the strong electron-withdrawing effect of the pyridine *N*-atom, which renders the proton more acidic, and therefore, the chemical shift for H_8_ was moved toward the downfield zone in the spectrum. PCM calculation did not take into account this effect, because the distances H_7_–N (3.36 Å) and H_8_–N (2.52 Å) remained almost constant with respect to gas phase calculation. The distribution of the electron density shown in Figure 4 indicated that the presence of the electro-withdrawing –C≡N group delocalized the electron distribution, thus decreasing the negative charge (in red color) on the double bond (–CH=CH–) and on the bonds of the phenyl ring with respect to 2-styrylpyridine. In the ***IIIa*** and ***IIIb*** molecules, proton H_8_ was more profoundly affected by the proximity of the lone electron pair of the *N*-atom, which was manifested as a shorter distance (H_8_–N = 2.52 Å), leaving the proton more labile or unshielded. This fact might explain the larger chemical shift value found with respect to the proton H_7_, located at a farther distance from the *N*-atom.

For molecule ***IIIc***, the behavior of protons H_7_ and H_8_ could be due to the A–π–A structure rather than by the presence of the lone electron pair of the *N-*atom, because the distances were longer: H_8_–N = 5.13 Å and H_7_–N = 4.82 Å. So, the influence of the *N*-atom in the *para*-position and the symmetry in the molecule played a more important role than the *N*–proton distances in explaining the calculated and measured chemical shifts. The electron density and chemical environment about both protons was very similar in the molecule, and therefore, the δ values were very similar.

From ^1^H NMR of the analogous halogen-substituted compounds, the principal signals for protons in the *trans* position of a double bond were reported at 7.604–7.550 ppm and 7.079–7.025 ppm for the *p*-fluoro-2-styrylpyridine, while for *p*-chloro-2-styrylpyridine, signals appeared at 7.294–7.240 and 7.033–6.979 [30]. These results are in agreement with our results for CN-substituted compounds ***IIIa*** and ***IIIb*** (Table 7). On the other hand, our results for the molecule ***IIIc*** are in good agreement with respect to previously reported data for 4-styrylpyridine, as well as for the Cl-substituted derivative. The chemical shifts for protons in the double bond were at 7.591–7.537 and 7.126–7.072 ppm [30].

In Table 7, the values of chemical shifts for the other protons theoretically characterized and reported, for 2-styrylpyridine (H5′ and H3′, error = 2.8%), ***IIIa*** (H3′, error = 4.8%), ***IIIb*** (H4′, error = 1.4%) and ***IIIc*** (H2 and H6, error = 11.7%) were in agreement with the experimental values obtained in the laboratory. These errors were increased by ~2% when the solvent effect was included, except for the molecule ***IIIb***, whose error value decreased in 0.4% by using the PCM calculation.

The theoretical ^1^H NMR data for the compounds were extremely useful for the assignment of the double signals in experimental spectra and above all for the assignments for the H_7_ and H_8_ positions, which in the experimental spectrum were not easily distinguished.

## 4. Experimental Section

### 4.1. Chemicals

3-Cyanobenzaldehyde, 2-picoline, 4-picoline, 2,6-lutidine, acquired from Aldrich Chemical Co. (Mexico city, Mexico), were purified before use. The IR spectra were recorded with a Vertex model 70 Bruker 750 FT-IR spectrophotometer by ATR. ^1^H (400 MHz) NMR spectra were obtained with a Varian NMR spectrometer using CDCl_3_ as solvent. UV-Vis absorption spectra were recorded at room temperature in CHCl_3_ with an Ocean Optics SD2000 spectrometer equipped with the light source UV-VIS DT 1000 CE from Analytical Instrument System, using 1 cm quartz cell.

### 4.2. Synthesis

#### *trans*-2-(*m*-Cyanostyryl)pyridine

3-Cyanobenzaldehyde (3.81 mmol) and 2-picoline (7.5 mmol) were mixed together. The mixture was magnetically stirred and refluxed for 30 h at 120 °C in the absence of any solvent or catalyst. The oily brown mixture was treated with a solution of 250 mL of 2 N NaOH with stirring for 24 h to yield a brown precipitate. The product was purified by recrystallization from cyclohexane.

#### *trans*-2-[3-Methyl-(*m*-cyanostyryl)]pyridine

3-Cyanobenzaldehyde (3.81 mmol) and 2,6-lutidine (3.37 mmol) were mixed together. The mixture was magnetically stirred and refluxed for 22 h at 140 °C in the absence of any solvent or catalyst. The oily brown mixture was treated with a EtOH:H_2_O solution of a 1:1 molar ratio to yield a brown precipitate. The product was purified by recrystallization with cyclohexane.

#### *trans*-4-(*m*′-Cyanostyryl)pyridine

3-Cyanobenzaldehyde (3.81 mmol) was mixed with 4-picoline (7.6 mmol). The oily brown mixture was magnetically stirred and refluxed for 30 h at 120 °C in the absence of any solvent or catalyst. The oily product was precipitated by using cyclohexane and by scraping with a spatula. The product was purified by recrystallization with cyclohexane.

## 5. Conclusions

We have successfully prepared three new conjugated styrylpyridine derivatives containing two aromatic rings, one of them a phenyl ring with a cyano group in the *m*-position and the others with a pyridine ring in either the *o*- or the *p*-position. These compounds were synthesized by Knoevenagel condensation between benzaldehydes with methylpyridines under green chemistry conditions. The structures were completely characterized by experimental and theoretical methods to obtain IR, UV-Vis and NMR ^1^H spectroscopy data.

Fully optimized geometries for the three molecules were obtained by DFT calculations showing planar structures, and the parameters were very similar to the X-ray data reported for the compounds 2- and 4-styrylpyridine. Solvent effects of CHCl_3_ were included with the PCM model for better spectroscopic characterizations. Two-dimensional conformational and population analyses show that different isomer states of ***IIIa***–***IIIc*** can exist with similar values of populations, while for the 2-styrylpyridine, its global and local minima can be clearly differentiated.

The theoretical UV-Vis, IR and NMR ^1^H data adequately reproduced the experimental data. Also, the theoretical study helped to identify clearly the characteristic vibration frequencies in the infrared spectra of the compounds. The assignment of characteristic normal vibrational modes as the δ(C–H) due to the protons of –CH=CH– in the *trans* configuration conjugated with aromatic rings and ν(C≡N) for compounds containing a cyano group conjugated with an aromatic ring were performed and adequately assigned experimentally, as well as theoretically.

The TD-DFT approach provided satisfactory insight into the main valence transitions identified experimentally from absorption measurements. The electron-withdrawing cyano group at the *meta*-position of the phenyl group had minimal influence on the overall electronic absorption of 2-sytyrylpyridine for ***IIIa*** and ***IIIb***, a result that is consistent with previous investigations with other electron-withdrawing groups, such as halogens (–Cl and –F). However, for molecule ***IIIc***, the position of the *N*-atom in the molecule in the *para*-position and the presence of the –C≡N substituent introduced a hypsochromic shift in the UV-Vis spectrum, which could be attributed to an A–π–A structure.

^1^H NMR chemical shifts for the protons attached in the *trans* position of a double bond appeared at 7.7–7.0 ppm as doublets, and the assignment was correctly made with the help of theoretical ^1^H NMR calculations.

The experimental and theoretical characterization carried out on the compounds gave evidence of their formation. In addition, from a theoretical viewpoint, the calculations described the molecular structures accurately and gave complete, as well as convincing, spectroscopic results that could be used to design new molecules with specific properties. In this form, the synthesis of model compounds could be predicted gaining insights into their structural properties to create polymeric materials prepared with D–π–A or A–π–A structures.

## Figures and Tables

**Table 1:** Conditions, yields and properties of the styrylpyridine-like model compounds (***IIIa***–***IIIc***).

Compound	Temp	Time	Yield	Appearance	Melting point	Solubility

	°C	h	%		°C	
***IIIa***	120	30	72	white powder	77–78	toluene, CHCl_3_, THF, acetone, EtOH, MeOH, DMSO
***IIIb***	140	22	59	beige powder	68–78	CHCl_3_, THF, acetone, MeOH, DMSO
***IIIc***	120	30	63	beige powder	63–65	hexane, cyclohexane, toluene, CHCl_3_, THF, acetone, EtOH, MeOH, DMSO

**Table 2:** Relative energies in (kJ mol^−1^) calculated at the B3LYP/6-311 + G(d,p) theory level and populations in (%) of the styrylpyridine-like model compounds (***IIIa***–***IIIc***) for different rotamers, with θ1 and θ2 dihedral angles in (°).

2-Styrylpyridine			*IIIa*		
	
θ1, θ2	Relative energy	Population	θ1, θ2	Relative energy	Population
0.0, 0.0	0.000	11.847	0.0, 0.0	0.000	6.854
0.0, 180.0	4.647	1.797	0.0, 180.0	6.459	0.076
30.0, 30.0	8.113	0.087	30.0, 30.0	7.903	0.064
60.0, 60.0	31.821	0.002	60.0, 60.0	31.532	0.001
90.0, 0.0	20.033	0.008	90.0, 0.0	19.088	0.003
90.0, 90.0	45.815	0.000	90.0, 90.0	45.658	0.000
90.0, 180.0	25.074	0.009	90.0, 180.0	24.916	0.003
180.0, 0.0	0.000	11.847	180.0, 0.0	0.630	5.254
180.0, 180.0	4.647	1.797	180.0, 180.0	5.277	1.231

***IIIb***			***IIIc***		
	
**θ1, θ2**	**Relative energy**	**Population**	**θ1, θ2**	**Relative energy**	**Population**

0.0, 0.0	0.000	6.581	0.0, 0.0	0.000	5.246
0.0, 180.0	6.091	0.011	0.0, 180.0	0.053	4.348
30.0, 30.0	7.850	0.025	30.0, 30.0	2.757	0.077
60.0, 60.0	31.296	0.007	60.0, 60.0	19.770	0.004
90.0, 0.0	19.114	0.005	90.0, 0.0	18.168	0.002
90.0, 90.0	45.290	0.001	90.0, 90.0	33.712	0.001
90.0, 180.0	24.627	0.013	90.0, 180.0	25.074	0.005
180.0, 0.0	0.525	5.541	180.0, 0.0	0.000	5.056
180.0, 180.0	5.094	0.897	180.0, 180.0	0.053	4.348

**Table 3:** Theoretical structural parameters of the equilibrium structures of the styrylpyridine-like model compounds (***IIIa***–***IIIc***) calculated at the B3LYP/6-311 + G(d,p) theory level. The numbering convention is shown in Figure 1. Internuclear distances in (Å), valence and dihedral angles in (°).

	*IIIa*		*IIIb*		*IIIc*	

Parameter	Gas	PCM	Gas	PCM	Gas	PCM
7–8	1.344	1.344	1.344	1.344	1.344	1.345
7–1	1.467	1.467	1.468	1.468	1.465	1.465
8–1′	1.465	1.465	1.464	1.465	1.466	1.466
4–3	1.397	1.397	1.339	1.402	1.335	1.338
3–2	1.330	1.332	1.335	1.337	1.392	1.391
2–1	1.347	1.349	1.346	1.346	1.402	1.403
3′–C	1.433	1.432	1.432	1.432	1.432	1.431
C≡N	1.156	1.156	1.155	1.156	1.155	1.156
1–7–8	124.1	124.5	124.1	124.5	126.5	126.1
2–1–7	118.5	118.7	118.4	118.7	119.3	119.3
7–8–1′	127.1	127.0	127.2	127.0	126.9	126.7
2′–3′–C	119.6	119.5	119.6	119.5	119.7	119.6
3′–C≡N	180.0	179.9	179.9	179.9	179.9	179.9
2–1–7–8	0.1	0.0	0.4	0.1	179.3	176.9
1–7–8–1′	180.0	180.0	180.0	180.0	180.0	180.0
7–8–1′–2′ a	0.4	0.3	0.7	0.2	179.2	176.6
C–3–2–1	---	---	179.9	180.0	---	---
1′–2′–3′–C	180.0	180.0	180.0	180.0	180.0	179.9

a 7–8–1′–6′ for molecule ***IIIc***.

**Table 4:** Calculated B3LYP/6-311 + G(d,p) and experimental harmonic frequencies in (cm^−1^) of the 2-styrylpyridine and ***IIIa***–***IIIc*** compounds.

	2-Styrylpyridine			*IIIa*			*IIIb*			*IIIc*		

Assignment	Gas	PCM	Exp a	Gas	PCM	Exp b	Gas	PCM	Exp b	Gas	PCM	Exp b
δ(C–H) out of plane in *trans* config.	981.5	982.5	985s	971.4	972.5	983 s	968.3	972.3	973s	970.3	973.0	975 s
ν(C=C)Py	1407.5	1405.7	1426 s	1410.0	1408.8	1431 s	1550.6	1546.7	1481 w	1390.2	1389.7	1418 s
ν(C=N)Py	1446.8	1446.5	1468 s	1457.6	1457.1	1476 s	1576.6	1574.2	1580 s	1471.6	1472.2	1481 s
[ν(C=N) + ν(C=C)]Py	1471.9	1470.6	1494 s	1544.0	1541.6	1491 w	------	------	------	1530.6	1526.4	1549 s
ν(C=N)Py + ν(C=C)Ph	1556.1	1553.7	1580 s	1576.9	1574.6	1584s	------	------	------	1578.0	1575.9	1594 s
ν(C=C)	1625.9	1624.1	1635 m	1628.3	1626.7	1638 m	1628.9	1626.8	1641 m	1628.2	1625.7	1640 m
ν(C≡N)	---	---	---	2250.5	2242.6	2232 s	2250.3	2242.5	2231 s	2252.3	2244.0	2230 s
ν(C–H)Py	3055.7	3061.4	3074 m	3034.2	3036.2	3066 m	3079.4	3081.3	3032 w	3043.1	3046.6	3065 m
ν(C–H)Ph	3078.8	3080.1	3141 w	3090.5	3092.0	3146 w	3089.7	3091.8	3079 m	3090.5	3093.3	3154 w
δ^−^_as_(CH_3_)	---	---	---	---	---	---	1020.9	1019.8	1036 m	---	---	---
δ_s_(CH_3_)	---	---	---	---	---	---	1360.8	1356.7	1374 m	---	---	---
δ^+^_as_(CH_3_)	---	---	---	---	---	---	1446.0	1439.6	1451 s	---	---	---
ν_s_(CH_3_)	---	---	---	---	---	---	2928.2	2926.9	2925 m	---	---	---
ν_as_(CH_3_)	---	---	---	---	---	---	2982.6	2980.0	2959 s	---	---	---

a Data IR(KBr) experimental in [27];

b IR(KBr) experimental data obtained in this work.

**Table 5:** Electronic transition, their assignments, the absorption maxima and oscillator strengths of the 2-styrylpyridine and ***IIIa***–***IIIc*** compounds calculated at the B3LYP/6-31G(d)//B3LYP/6-311 + G(d,p) theory level.

	Electronic transition	Absorption maxima (nm (eV))		Oscillator strengths		MO/Character (% Coefficient)	Experimental (nm (eV))
		Gas	PCM	Gas	PCM		
2-styrylpyridine	S_0_–S_1_	311.6 (3.98)	323.3 (3.83)	0.8922	1.0597	HOMO→LUMO (98%)	311.0 (3.99)
	S_0_–S_2_	291.2 (4.26)	288.1 (4.30)	0.0014	0.0014	HOMO-2→LUMO (97%)	
	S_0_–S_3_	270.9 (4.58)	271.6 (4.57)	0.0030	0.0049	HOMO-1→LUMO (74%) + HOMO→LUMO+2 (25%)	

***IIIa***	S_0_–S_1_	309.3 (4.01)	320.9 (3.86)	0.9656	1.1193	HOMO→LUMO (98%)	309.6 (4.01)
	S_0_–S_2_	298.4 (4.16)	300.5 (4.13)	0.0010	0.0133	HOMO-1→LUMO (96%)	
	S_0_–S_3_	295.7 (4.19)	295.2 (4.20)	0.0116	0.0012	HOMO→LUMO + 1 (86%) + HOMO-2→LUMO (12%)	

***IIIb***	S_0_–S_1_	312.7 (3.96)	324.1 (3.83)	0.9432	1.0916	HOMO→LUMO (97%)	312.0 (3.98)
	S_0_–S_2_	302.7 (4.10)	302.8 (4.10)	0.0010	0.0141	HOMO-1→LUMO (96%)	
	S_0_–S_3_	297.7 (4.16)	300.3 (4.13)	0.0088	0.0012	HOMO→LUMO + 1 (82%) + HOMO-2→LUMO (14%)	

***IIIc***	S_0_–S_1_	307.2 (4.04)	318.3 (3.89)	0.7615	0.9422	HOMO→LUMO (98%)	293.6 (4.23)
	S_0_–S_2_	285.4 (4.34)	296.9 (4.18)	0.1545	0.1452	HOMO→LUMO + 1 (71%) + HOMO-3→LUMO (24%)	
	S_0_–S_3_	309.0 (4.01)	288.1 (4.30)	0.0021	0.0018	HOMO-1→LUMO (93%)	

**Table 6:** Orbital energies (au) and Gap energies (eV) of the 2-styrylpyridine and ***IIIa***–***IIIc*** compounds calculated at the B3LYP/6-31G(d)//B3LYP/6-311 + G(d,p) theory level.

	Є_HOMO-1_		Є_HOMO_		Є_LUMO_		Є_LUMO+1_		Gap energy	

	Gas	PCM	Gas	PCM	Gas	PCM	Gas	PCM	Gas	PCM
2-stypy	−0.2625	−0.2654	−0.2201	−0.2234	−0.0714	−0.0747	−0.0340	−0.0361	4.04	4.04
***IIIa***	−0.2742	−0.2737	−0.2366	−0.2340	−0.0867	−0.0841	−0.0638	−0.0647	4.08	4.08
***IIIb***	−0.2702	−0.2698	−0.2338	−0.2317	−0.0848	−0.0828	−0.0629	−0.0643	4.05	4.05
***IIIc***	−0.2727	−0.2749	−0.2491	−0.2429	−0.0979	−0.0920	−0.0684	−0.0646	4.11	4.10

**Table 7:** Theoretical and experimental ^1^H NMR chemical shifts (ppm) of the 2-styrylpyridine and ***IIIa***–***IIIc*** compounds calculated at the B3LYP/6-311 + G(2d,p)//B3LYP/6-311 + G(d,p) theory level. All values are referenced to the chemical shift of TMS computed at the same theory level.

Chemical shifts, δ (ppm)							

	2-styrylpyridine				*IIIa*		
			
	Gas	PCM	Exp a		Gas	PCM	Exp b
H3 (d, 1H)	8.964	8.993	8.653–8.639	H3 (d, 1H)	8.916	8.942	8.620–8.609
H6′, H2′ (m, 2H)	8.148–7.645	8.285–7.752	7.630–7.613	H2′ (s, 1H)	8.425	8.582	7.818
H7 (d, 1H)	7.418	7.594	7.240–7.186	H7 (m, 2H)	7.462	7.629	7.204–7.164
H8 (d, 1H)	8.461	8.362	7.708–7.654	H8 (d, 2H)	8.406	8.335	7.632–7.592
H5′, H3′ (m, 3H)	7.593–7.544	7.716–7.684	7.416–7.310	H5′ (m, 1H)	7.818	8.021	7.488–7.442

	***IIIb***				***IIIc***		
			
	**Gas**	**PCM**	**Exp**^b^		**Gas**	**PCM**	**Exp**^b^

H (s, 3H, CH^3^)	2.714	2.704	2.599	H3, H5 (d, 2H)	8.952	8.936	8.605–8.585
H2′ (s, 2H)	8.419	8.577	7.827	H2′ (s, 1H)	7.783	7.971	7.791
H7 (d, 1H)	7.408	7.569	7.220–7.180	H7 (d, 1H)	7.574	7.746	7.074–7.020
H8 (d, 1H)	8.379	8.304	7.605–7.564	H8 (d, 1H)	7.365	7.525	7.261–7.207
H4′ (d, 1H)	7.677	7.837	7.796–7.777	H2, H6 (m, 2H)	8.220	8.444	7.365–7.350

a Experimental values from [24] obtained to 400 MHz in CDCl_3_;

b Experimental values in this work obtained to 300 MHz in CDCl_3_.
